# Rebleeding Rate and Risk Factors for Rebleeding after Device-Assisted Enteroscopy in Patients with Obscure Gastrointestinal Bleeding: A KASID Multicenter Study

**DOI:** 10.3390/diagnostics12040954

**Published:** 2022-04-11

**Authors:** Yuna Kim, Jae-Hyun Kim, Eun-Ae Kang, Soo-Jung Park, Jae-Jun Park, Jae-Hee Cheon, Tae-Il Kim, Jihye Park, Seong-Ran Jeon

**Affiliations:** 1 Department of Internal Medicine and Institute of Gastroenterology, Yonsei University College of Medicine, Seoul 03722, Korea; sadts@yuhs.ac (Y.K.); eakang@yuhs.ac (E.-A.K.); sjpark@yuhs.ac (S.-J.P.); jaejpark@yuhs.ac (J.-J.P.); geniushee@yuhs.ac (J.-H.C.); taeilkim@yuhs.ac (T.-I.K.); 2 Division of Gastroenterology, Department of Internal Medicine, Kosin University College of Medicine, Busan 49267, Korea; kjh8517@daum.net; 3 Institute for Digestive Research, Digestive Disease Center, Soonchunhyang University College of Medicine, Seoul 04401, Korea

**Keywords:** enteroscopy, obscure gastrointestinal bleeding, rebleeding, risk factor

## Abstract

Introduction: The impact of device-assisted enteroscopy (DAE) on long-term rebleeding in patients with obscure gastrointestinal bleeding (OGIB) exhibiting detectable small-bowel lesions remains unclear. We investigated the long-term rebleeding rate and predictive factors for DAE in patients with OGIB. Method: Patients with OGIB with small bowel lesions detected through DAE were enrolled at three Korean tertiary hospitals. Predictive risk factors associated with rebleeding were analyzed using the Cox regression analysis. Results: From April 2008 to April 2021, 141 patients were enrolled, including 38 patients (27.0%) with rebleeding. The rebleeding rates at 1, 2, and 3 years were 25.0%, 29.6%, and 31.1%, respectively. The Cox regression analysis revealed that multiple small-bowel lesions (hazard ratio [HR]: 2.551, 95% confidence interval [CI]: 1.157–5.627, *p* = 0.020), the need for more than five packed red blood cells (RBC) transfusions (HR: 2.704, 95% CI: 1.412–5.181, *p* = 0.003), and ulcerative lesions (HR: 1.992, 95% CI: 1.037–3.826, *p* = 0.039) were positively associated with rebleeding. Therapeutic interventions for patients with detectable lesions, overt bleeding (vs. occult bleeding), comorbidities, and medications were not associated with rebleeding. Conclusion: More than 25% of patients with OGIB having detectable small-bowel lesions had rebleeding. Patients with multiple lesions, a requirement of more than five packed RBC transfusions, and ulcerative lesions were associated with a higher risk of rebleeding.

## 1. Introduction

Obscure gastrointestinal bleeding (OGIB) is defined as gastrointestinal (GI) bleeding from a source that remains uncertain after upper and lower gastrointestinal endoscopy [1]. OGIB accounts for 5–10% of all cases of GI bleeding and it is divided into two types: occult and overt-OGIB [2,3]. Many of these cases are caused by small intestinal lesions [4].

Because of its high diagnostic yield and non-invasiveness, capsule endoscopy (CE) is recommended as the first-line examination for small-bowel assessment [5,6]. In overt-OGIB, the European Society of Gastrointestinal Endoscopy (ESGE) recommends CE as soon as possible within 14 days after a bleeding episode [4].

Device-assisted enteroscopy (DAE), which encompasses spiral, single-balloon endoscopy (SBE), and double-balloon enteroscopy (DBE), may be the first-line therapeutic procedure following a positive CE in an OGIB setting [7,8,9]. DAE is most often performed following a less invasive and simple small bowel investigation such as CE, since it allows the determination of the bleeding site and, consequently, a better approach in terms of route and therapeutic planning [10]. According to the current literature, the diagnostic yield of DAE for OGIB is around 60%, which is higher after a positive CE when compared to DAE performed after a negative CE [11]. DAE enables effective therapeutic interventions such as argon plasma coagulation or hemostatic clipping [12]. Although DAE is appropriate for small bowel evaluation, its usage is restricted due to prolonged duration, technical complexity, and the requirement for extensive equipment and several assistants [13].

The diagnosis and management of OGIB have substantially improved owing to the availability of CE and DAE; however, rebleeding remains a significant issue [14]. Furthermore, the advantages of DAE treatment remain debatable. Following the initial DAE treatment, Ponte et al. reported a 52.6% rebleeding rate after a 24-month follow-up [15]. OGIB rebleeding is associated with high morbidity, suggesting that it should be monitored regularly. On the other hand, regularly following patients with low rebleeding risk is neither useful to the patient nor an optimal use of healthcare resources. However, the risk factors associated with rebleeding after DAE are not fully understood. Therefore, we aimed to identify the risk factors for rebleeding after DAE and the long-term rebleeding rate in patients with OGIB in a multicenter study.

## 2. Methods

### 2.1. Patients and Study Design

**Figure 1:**
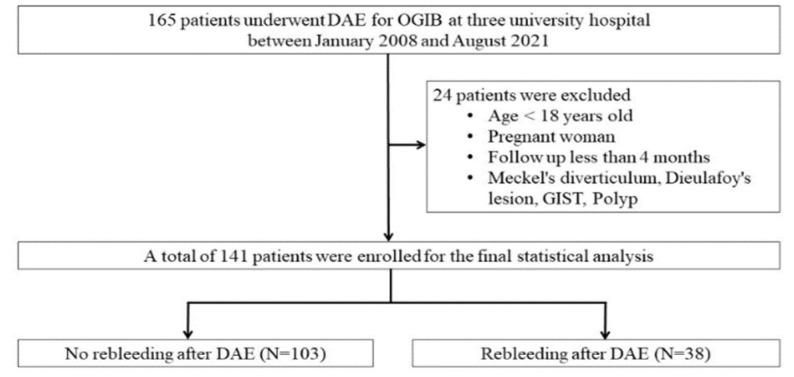
Flow chart of selecting the study population. Among 165 patients that underwent DAE for OGIB, 24 were excluded according to the exclusion criteria. Finally, 141 patients from three Korean institutes were selected for the statistical analysis.

We retrospectively reviewed the medical records of 165 patients who underwent DAE for OGIB at three university hospitals between January 2008 and August 2021. Of these 165 patients, 141 patients who had been diagnosed with OGIB and small-bowel lesions with DAE were enrolled in this study (Figure 1). Patients who were not diagnosed with small-bowel lesions by the enteroscopy and those with small bowel tumors were excluded. Patients under 18 years of age and pregnant women were excluded from the analysis. We also excluded patients with a follow-up time of fewer than 4 weeks or those with insufficient data during the follow-up period. Patient data and follow-up information were collected from hospital electronic medical charts. The variable data included age, sex, comorbidity, medication use (anticoagulants, antiplatelets, and non-steroidal anti-inflammatory drugs), presentation of OGIB (overt or occult), history of previous overt GI bleeding, hemoglobin level, type of specific treatment and time interval to the rebleeding event after DAE. The patients were divided into two groups, one with rebleeding and one without rebleeding after DAE, and the risk factors for rebleeding were investigated. Due to the retrospective nature of this study, the need for written informed consent was waived. The study protocol was approved by the Institutional Review Board and Hospital Research Ethics Committee of each facility.

### 2.2. Device Assisted Enteroscopy

A DBE (EN-450P5, T5 or EN-530T; Fujinon Inc., Saitama, Japan) and anf SBE (SIF-Q180; Olympus America Inc., Center Valley, PA, USA), both of which are available in South Korea, were used for enteroscopic examinations. The route of DAE insertion was determined based on the location of the bleeding, according to the results of previous examinations. All procedures were performed with a fluoroscopy unit, with patients undergoing conscious to deep sedation (established by endoscopists) in accordance with each center’s sedation protocols.

### 2.3. Therapeutic Strategy

Therapeutic management was based on the etiology of bleeding, and the patient’s condition. Treatment was classified into two groups. The group included patients whose lesions had undergone endoscopic hemostasis with DAE. This group received argon plasma coagulation, hemostatic clipping, or both. The other group included patients who did not undergo endoscopic hemostasis with DAE. Regardless of the group, a few patients eventually required surgery or embolization. If needed, patients received additional medical treatments, including mucoprotective agents such as rebamipide, blood transfusions, and iron supplementation.

### 2.4. Follow Up and Outcomes

Recurrence of bleeding following DAE and the date of rebleeding were obtained from the patients’ electronic medical data. The duration of follow-up was defined as the interval between the DAE study and the date of rebleeding, or the last follow-up visit. Patients with a follow-up period of fewer than four weeks were excluded, as were those with insufficient data to assess a rebleeding event during the follow-up. The primary outcome of this study was the recurrent bleeding rate after DAE. Rebleeding was defined as recurrent bleeding episodes with overt bleeding defined by the presence of melena or hematochezia with a decrease in hemoglobin levels greater than 2 g/dL, or occult bleeding defined by an unexplained decrease in hemoglobin levels greater than 2 g/dL in the absence of melena or hematochezia without bleeding focus in the repeat conventional endoscopies. The secondary outcomes were risk factors for rebleeding following DAE.

### 2.5. Statistical Analysis

Patient characteristics were described as median and range for continuous variables, and percentages for categorical variables. Student’s *t*-test or Mann–Whitney U test was used to compare quantitative variables. To compare qualitative variables, the chi-squared test or Fisher’s exact test was used. The Kaplan–Meier method was used to estimate the rebleeding rate, which was then compared using the log-rank test. To identify the risk factors independently associated with rebleeding after DAE, multivariate analysis using the Cox regression model was performed on variables that were significantly associated with rebleeding after DAE in the univariate analysis (*p* < 0.05). The results are reported as hazard ratios (HRs) with a confidence interval (CI) of 95%. IBM SPSS Statistics software package version 23.0.0.0 (IBM SPSS Inc., Chicago, IL, USA) was used for all statistical analyses.

## 3. Results

### 3.1. Patient Characteristics

A total of 141 patients with OGIB who underwent DAE were analyzed. The baseline characteristics of the study population at the time of the DAE are described in Table 1. Of the 141 patients, 90 patients (63.8%) were men. The median age and basal metabolic index (BMI) were 69.0 years (Inter-quartile range [IQR] 55.0–80.0) and 22.8 kg/m^2^ (IQR 19.6–25.3), respectively. A total of 128 patients (90.8%) were diagnosed with overt OGIB, while 13 patients (9.2%) were diagnosed with occult OGIB. A total of thirty-seven patients (35.2%) had a history of previous overt gastrointestinal bleeding and 54 patients (38.3%) had used medications associated with bleeding tendencies, such as aspirin, clopidogrel, warfarin, and non-vitamin K antagonist oral anticoagulants. A total of eighty-eight patients (62.4%) had comorbidities including hypertension, diabetes mellitus, chronic heart disease, valve disease, chronic kidney disease, and chronic liver disease. SBE was performed in 28 patients (19.9%), whereas DBE was performed in 113 patients (80.1%). DAE was performed within 72 h of a bleeding event in 20 patients (14.2%). The median hemoglobin level before DAE was 8.2 g/dL (IQR 6.9–9.6).

Angiodysplasia was observed in 66 patients (46.8%), erosion in 43 patients (30.5%), and ulcers in 60 patients (42.6%). These lesions were located in the jejunum in 72 patients (51.1%), ileum in 52 patients (36.9%), and in both the jejunum and ileum in 17 patients (12.1%). A single lesion was observed in 52 patients (36.9%), whereas multiple lesions were observed in 89 patients (63.1%). Hemostasis was attempted in 93 patients (66.0%). Argon plasma coagulation were used to treat 42 patients (45.2%), hemostatic clipping was performed in 31 patients (33.3%), and both argon plasma coagulation and hemostatic clipping was used to treat 20 patients (21.5%). During the follow-up period, 9 patients (6.4%) required embolization, and 13 patients (9.2%) required eventual surgery. During the hospital stay, 41 patients (29.1%) required more than five units of red blood cell (RBC) transfusion. No cases of morbidity were associated with the enteroscopic procedure.

### 3.2. Comparison of Patients Who Had Rebleeding and Those Who Did Not

Rebleeding occurred in 38 patients (27.0%) during a median follow-up of 26.0 months (IQR 2.5–76.0 months). Baseline characteristics were compared between patients who had rebleeding after DAE (*n* = 38, 27.0%) and those who did not have rebleeding after DAE (*n* = 103, 73.0%) (Table 1). Patients with rebleeding experienced unfavorable events when compared with patients without rebleeding, with significantly higher frequencies of ulcers (57.9% vs. 36.9%, respectively; *p* = 0.025) and multiple numbers of lesions (78.9% vs. 57.3%, respectively; *p* = 0.018). The requirement for an RBC transfusion of more than five units was also significantly higher in patients with rebleeding than in patients without rebleeding (44.7% vs. 23.3%, respectively; *p* = 0.013). On the other hand, comorbidities including hypertension, diabetes, cardiac disease, liver cirrhosis, and chronic kidney disease did not show statistically significant differences between the two groups. Although there was no significant difference in hospital days between the two groups, the rebleeding group’s median hospital day was longer than the non-rebleeding group’s. The median hospital days were 11.0 days (IQR 8.0–16.0) and 13.5 days (IQR 8.8–23.2) in the no-rebleeding group and rebleeding group, respectively (*p* = 0.163). The other variables were also comparable between the two groups (all *p* > 0.05).

### 3.3. Risk Factors for Rebleeding

In the univariate analysis, the presence of ulcerative lesions, two or more multiple lesions, and the requirement for RBC transfusion of more than five units were significantly associated with an increased risk of rebleeding after DAE (all *p* < 0.05). Subsequent multivariate analyses also revealed that the presence of ulcerative lesions (HR = 2.594, 95% CI, 1.145–5.874; *p* = 0.022), two or more multiple lesions (HR = 3.231, 95% CI, 1.263–8.269; *p* = 0.014), and requirement for RBC transfusion of more than five units (HR = 3.700, 95% CI, 1.152–8.821; *p* = 0.003) independently predicted an increased risk of rebleeding following DAE (Table 2).

**Figure 2:**
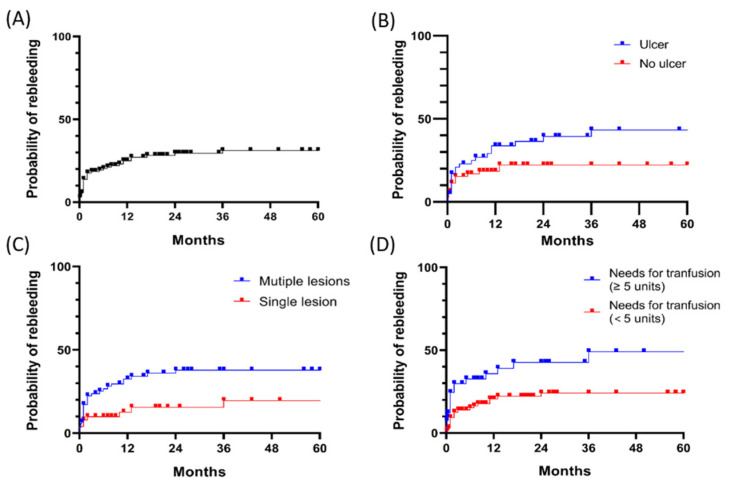
Cumulative incidence of rebleeding and associated factors. (**A**) All rebleeding; (**B**) Rebleeding stratified by presence of ulcer; (**C**) Rebleeding stratified by the number of lesions; (**D**) Rebleeding stratified by requirements of more than five units of RBC transfusion.

The cumulative rebleeding rates and associated factors are shown in Figure 2. The rebleeding rates in the entire study population (*n* = 141) were 25.0%, 29.6%, and 31.1% at 12, 24, and 36 months, respectively (Figure 2A). The cumulative rebleeding rates of patients with ulcerative lesions, those with two or more lesions, and those who required RBC transfusion of more than five units were significantly higher than those of their counterparts (*p* = 0.011, *p* = 0.030, and *p* = 0.017 by log-rank test, respectively) (Figure 2B–D). However, overt vs. occult OGIB, time to enteroscopy from a bleeding event, and hemostasis treatment were not associated with rebleeding in patients with OGIB.

## 4. Discussion

DAE has a high diagnostic yield and allows endoscopic hemostasis in patients with OGIB. The diagnostic yield of DAE is reported to be from 60 to 80% and the therapeutic yield of DAE is reported to be from 40 to 73% [16,17]. In 40–50% of patients, findings from DAE influence the treatment strategies [18]. Recently, urgent DAE (<72 h) of OGIB patients has been reported to improve the diagnostic rate compared to non-urgent DAE, and the treatment rate was also improved [19]. Despite the advancement of DAE in the diagnosis and treatment of OGIB, rebleeding events still occur and remain an unresolved concern. Therefore, identifying the risk factors related to rebleeding rates is important and could help in the management of patients with OGIB. In this study, we investigated the long-term rebleeding rate and risk factors for rebleeding after detecting small bowel lesions using DAE in patients with OGIB. To our knowledge, this is the largest study to date to evaluate the risk factors of rebleeding following DAE in patients with OGIB.

The rebleeding rate in OGIB cases has been assessed in different studies; however, the data vary. In the present study, we observe a rebleeding rate of 29.6% at 24 months, which is similar to previous studies (rebleeding rate of 16.7–42.9%) [20,21,22,23]. There are at least two possible explanations for varying rebleeding rates. First, it has been observed that the incidence of coronary heart disease has increased significantly and that the use of anticoagulants and antiplatelets is increasing. In addition, the elderly population with chronic diseases, such as chronic kidney disease or liver cirrhosis, which have a higher risk of bleeding, is growing. Second, there is heterogeneity in the diagnostic yields of DAE among different countries, which could be due to differences in patient composition, endoscopic diagnostic criteria, and endoscopist experience [24]. Therefore, we considered that the profile of each study subject may differ from that of other studies.

Our findings demonstrate that patients with an ulcerative lesion, two or more multiple lesions, or who required more than five units of RBC transfusions were at an increased risk of rebleeding after the detection of small-bowel lesions using DAE. These findings are consistent with previous research on rebleeding risk factors. Several previous studies have reported that ulcerative lesions, angioectasias, tumors, diverticulae, and nonsteroidal anti-inflammatory drug use are potential causes of small-bowel lesions in OGIB [25,26]. According to Shinozaki et al., multiple lesions, along with the need for a large transfusion, significantly increase the risk of rebleeding in patients with small bowel lesions [20]. According to another recent study by Grooteman et al., only multiple lesions increase the risk of rebleeding following DAE detection of angiodysplasia [27]. Perz et al. suggested that overt bleeding, multiple lesions, cardiac disease, and liver cirrhosis were predictors of rebleeding after DAE for small bowel vascular lesions [28]. However, in our study, each comorbidity, including hypertension, diabetes, cardiac disease, liver cirrhosis, and chronic kidney disease, did not have a statistically significant impact on the rebleeding rate. This is explicable in two ways. First, unlike our study, including both ulcer, erosion, and angiodysplasia, many other studies have only targeted vascular lesions in the small bowel such as angiodysplasia. Other studies have established that the risk factors for angiodysplasia in the GI tract include old age, liver cirrhosis, and chronic kidney disease. Second, 49, 8, and 32 of the patients in this study had cardia disease, liver cirrhosis, or chronic kidney disease, respectively. The nature of the retrospective study, as well as the selection bias, may have contributed to the heterogeneous results.

In a previous study, on the other hand, endoscopic hemostasis with DAE did not significantly affect the rebleeding rate in patients with small bowel angioectasia bleeding [29]. Our study also reported that enteroscopic hemostasis did not appear to be a statistically significant risk factor for rebleeding in patients with OGIB. This could be for several reasons. In our study, multiple lesions were detected in 63.1 percent of patients. Because few patients received complete DAE using a one-side approach, it is possible that some small bowel lesions were missed in the absence of treatment. Several studies have suggested that APC of endoscopically accessible small bowel lesions may reduce the need for transfusion, however many patients continue to bleed [11,30]. Thus, detecting all lesions and performing enteroscopic hemostasis are impractical, and interpreting the rebleeding rate could be misleading. As a result, regardless of hemostatic treatment, close monitoring and long-term follow-up of patients at high risk may be necessary.

We are also aware of several limitations of this study that need to be addressed. First, because this was a retrospective study with selection bias, the results cannot be generalized despite our efforts to recruit many patients through a multicenter study. Second, the follow-up times varied greatly, which might have influenced the rebleeding rate. Third, we could not assess the impact of therapeutic interventions, such as medications for reducing the risk of rebleeding, which may have compromised the results of the study. Finally, ulcerative or multiple lesions and the need for massive RBC transfusions are already determining factors that cannot be modified. Nevertheless, the strengths of this study included the long follow-up time (median 26 months) and the risk factors being evaluated in multivariate analyses. Our findings reveal the need to screen patients, particularly those with ulcerative or multiple lesions, and those who required RBC transfusions of more than five units to identify patients with a high risk of rebleeding.

In conclusion, patients with an ulcerative lesion, two or more multiple lesions, or who required more than five units of RBC transfusions, had a greater risk of rebleeding. We report three important risk indicators that might help clinicians predict cases of rebleeding as well as other clinical outcomes such as duration of stay and mortality in patients with small-bowel bleeding. OGIB rebleeding is high risk; therefore, it should be monitored regularly. Larger scale studies on DAE are required to validate the findings of the present study.

## Figures and Tables

**Table 1:** Baseline characteristics.

Variables	Total	No Rebleeding after Enteroscopy	Rebleeding after Enteroscopy	* *p* Value
	(*n* = 141)	(*n* = 103)	(*n* = 38)	
Age (years), median (IQR)	69.0 (55.0–80.0)	69.0 (55.0–80.0)	71.0 (56.0–81.0)	0.379
Sex (male), *n* (%)	90 (63.8)	70 (68.0)	20 (52.6)	0.093
Body Mass Index (kg/m^2^), median (IQR)	22.8 (19.6–25.3)	23.2 (19.7–25.4)	21.4 (19.1–24.9)	0.379
Bleeding type, *n* (%)				0.324
Overt OGIB/Occult OGIB	128 (90.8)/13 (9.2)	92 (89.3)/11 (10.7)	36 (94.7)/2 (5.3)	
Previous overt bleeding history, *n* (%)	37 (35.2)	25 (23.1)	12 (31.6)	0.382
History of medications associated with bleeding tendency ^†^, *n* (%)	54 (38.3)	38 (36.9)	16 (42.1)	0.572
Presence of comorbidities ^§^, *n* (%)	88 (62.4)	62 (60.2)	26 (68.4)	0.371
Type of Enteroscopy, *n* (%)				0.531
Single balloon/Double balloon	28 (19.9)/113 (80.1)	20 (19.8)/83 (80.6)	8 (21.1)/30 (78.9)	
Time to enteroscopy from bleeding event (<3 days), *n* (%)	20 (14.2)	16 (15.5)	4 (10.5)	0.521
Hemoglobin (g/dL) before enteroscopy, median (IQR)	8.2 (6.9–9.6))	8.5 (7.2–9.8)	8.0 (6.3–8.6)	0.462
Impression, *n* (%)				
Angioectasia	66 (46.8)	47 (45.6)	19 (50.0)	0.645
Erosion	43 (30.5)	32 (31.1)	11 (28.9)	0.808
Ulcer	60 (42.6)	38 (36.9)	22 (57.9)	0.025
Location, *n* (%)				
Jejunum	72 (51.1)	55 (53.4)	17 (44.7)	0.588
Ileum	52 (36.9)	39 (37.9)	13 (34.2)	0.147
Jejunum and ileum, both	17 (12.1)	9 (8.7)	8 (21.1)	0.251
Number of lesions (≥2), *n* (%)	89 (63.1)	59 (57.3)	30 (78.9)	0.018
Enteroscopic therapy, *n* (%)	93 (66.0)	70 (68.0)	23 (60.5)	
Argon plasma coagulation	42 (45.2)	29 (28.1)	13 (34.2)	0.485
Hemostatic clipping	31 (33.3)	25 (24.2)	6 (15.8)	0.281
Argon plasma coagulation and hemostatic clipping, both	20 (21.5)	13 (12.6)	7 (18.4)	0.381
Needs for embolization	9 (6.4)	5 (4.9)	4 (10.5)	0.222
Needs for surgery	13 (9.2)	7 (6.8)	6 (15.8)	0.101
Needs for RBC transfusion (≥5 units), *n* (%)	41 (29.1)	24 (23.3)	17 (44.7)	0.013

* *p* value for comparing patients with rebleeding and patients without rebleeding. ^†^ Medications include aspirin, clopidogrel, warfarin and non-vitamin K antagonist oral anticoagulant. ^§^ Comorbidities include hypertension, diabetes mellitus, chronic heart disease, valve disease, chronic kidney disease, and chronic liver disease. IQR, interquartile range; OGIB, obscure gastrointestinal bleeding.

**Table 2:** Factors associated with rebleeding following endoscopic therapy of small bowel lesions with device-assisted enteroscopy.

Variables	Univariate Analysis			Multivariate Analysis		
	HR	95% CI	* *p* Value	HR	95% CI	* *p* Value
Age (years)	0.990	0.987–1.018	0.950			
Sex (male)	0.605	0.320–1.144	0.122			
Body Mass Index (≥23 kg/m^2^)	0.533	0.260–1.095	0.087			
Over OGIB (vs. occult OGIB)	2.082	0.501–8.652	0.313			
Previous overt bleeding history	1.737	0.871–3.463	0.117			
History of medications associated with bleeding tendency ^†^	1.161	0.609–2.211	0.650			
Presence of comorbidities ^§^	1.274	0.643–2.527	0.487			
Double balloon enteroscopy (vs. single balloon enteroscopy)	2.401	0.735–7.846	0.147			
Time to enteroscopy from bleeding event (≤3 days)	0.634	0.159–1.455	0.389			
Hemoglobin (g/dL) before enteroscopy	0.824	0.691–0.982	0.080			
Impression						
Angiodysplasia	1.277	0.676–2.414	0.451			
Erosion	0.869	0.431–1.752	0.694			
Ulcer	2.352	1.102–5.020	0.027	2.594	1.145–5.874	0.022
Location						
Jejunum	1.000	reference	0.192			
Ileum	1.163	0.565–2.396	0.682			
Jejunum and ileum	2.160	0.932–5.008	0.073			
Number of lesion (≥2)	2.797	2.169–6.690	0.021	3.231	1.263–8.269	0.014
Enteroscopic therapy	0.727	0.379–1.396	0.338			
Needs for RBC transfusion (≥5 units)	2.665	1.214–5.847	0.015	3.700	1.152–8.821	0.003

* *p* value for comparing patients with rebleeding and patients without rebleeding. ^†^ Medications include aspirin, clopidogrel, warfarin, and non-vitamin K antagonist oral anticoagulant. ^§^ Comorbidities include hypertension, diabetes mellitus, chronic heart disease, valve disease, chronic kidney disease, and chronic liver disease. OGIB, obscure gastrointestinal bleeding; HR, hazards ratio; CI, confidence interval.

## Data Availability

The data that support the findings of this study are available from the corresponding author upon reasonable request.
